# CDK2 inhibition sensitizes anthracycline-induced immunogenic cell death and enhances the efficacy of anti-PD-1 therapy

**DOI:** 10.3389/fimmu.2025.1570040

**Published:** 2025-06-10

**Authors:** Yu Chen, Wancheng Liu, Qiaomei Cai, Chaohu Pan, Zhenghao Yin, Yijiao Tang, Zhixu He, Genhong Cheng, Liping Shu

**Affiliations:** ^1^ School of Basic Medicine, Affiliated Hospital of Guizhou Medical University, Guizhou Medical University, Guiyang, China; ^2^ Department of Clinical Laboratory, Qilu Hospital of Shandong University, Jinan, Shandong, China; ^3^ Department of Maxillofacial and Otorhinolaryngological Oncology, Tianjin Medical University Cancer Institute & Hospital, National Clinical Research Center for Cancer, Tianjin’s Clinical Research Center for Cancer, Key Laboratory of Basic and Translational Medicine on Head & Neck Cancer, Tianjin, Key Laboratory of Cancer Prevention and Therapy, Tianjin, China; ^4^ National Clinical Research Center for Infectious Diseases, Shenzhen Third People’s Hospital, Southern University of Science and Technology, Shenzhen, China; ^5^ Key Laboratory of Adult Stem Cell Translational Research, Chinese Academy of Medical Sciences, Guiyang, China; ^6^ Department of Microbiology, Immunology & Molecular Genetics, University of California, Los Angeles, Los Angeles, CA, United States

**Keywords:** immunogenic cell death, CDK2, anti-PD-1 therapy, type-1 interferon response, MTX

## Abstract

**Introduction:**

CDK2 (Cyclin-dependent kinase 2) is an oncogenic cyclin-dependent kinase with potent mitogenic and immunosuppressive functions. Despite extensive research on CDK2 inhibitors, the lack of selectivity has made it unclear whether CDK2 inhibition specifically facilitate immunogenic cell death.

**Methods:**

We used CRISPR-Cas9 system to generate *Cdk2^-/-^* MCA205 cells. Tumor cells were inoculated subcutaneously into mice while administering MTX (mitoxantrone) or anti-PD-1 antibodies treatment to observe tumor growth curves. Next, immune cell infiltration in tumor microenvironment was detected by immunofluorescence. Furthermore, apoptosis pathway was evaluated by flow cytometry and western blot. The hallmarks of immunogenic cell death were detected by flow cytometry, ELISA or qRT-PCR.

**Results:**

We found that mice bearing *Cdk2^-/-^* cancer cells exhibit slower tumor growth than WT cells after anthracycline analogue MTX treatment, and this phenomenon is dependent on the immune system. Furthermore, our data exhibits that *Cdk2^-/-^* cancer cells treated with MTX trigger a more robust immunostimulatory responses than WT cells, including apoptosis stress response, surface calreticulin expression, endoplasmic reticulum stress response, HMGB1 (High Mobility Group Box 1) release, and type-1 interferon response.

**Discussion:**

This study not only suggests that CDK2 inhibition improves the outcome of chemotherapy by enhancing the type-1 interferon response but also investigates the synergistic effects of CDK2 inhibition with MTX or anti-PD-1 antibodies in immunocompetent mice.

## Introduction

The antitumor immune response is shaped by a complex interplay within the tumor microenvironment, encompassing TILs (tumor-infiltrating lymphocytes), PD-L1 (Programmed Death-Ligand 1) expression, mutational burden, neoantigen presentation, and an IFN-γ(interferon-γ) gene signature (1–4). Despite the therapeutic potential of anti-PD-1 therapy, tumors that lack effective antigen presentation or adequate T cell infiltration remain largely unresponsive, underscoring the necessity for complementary strategies to modulate the immunosuppressive tumor environment (5). Inducing ICD (immunogenic cell death) within tumors represents a strategic approach, characterized by the release of DAMPs (damage-associated molecular patterns) that stimulate immune activity (6–9). For example, ATP (Adenosine Triphosphate) engages purinergic receptors attracting myeloid cells into the tumor bed (7, 10). The ‘eat-me’ signal calreticulin translocates to the plasma membrane, facilitating tumor antigen transfer to dendritic cells (11, 12). HMGB1 activates toll-like receptor 4 on dendritic cells, promoting their maturation (13). The type-1 interferon response leads to autocrine activation of IFNAR (interferon α/β receptors), resulting in CXCL9 (C-X-C motif chemokine ligand 9) and CXCL10 (C-X-C motif chemokine ligand 10) production, which subsequently stimulates T lymphocyte infiltration into the tumor (14). Cytotoxic agents such as anthracycline analogue MTX, oxaliplatin, radiation therapy, and oncolytic viruses have been shown to induce ICD, thereby enhancing tumor immunogenicity and promoting the recruitment and activation of APCs (antigen-presenting cells) and T cells (8, 14–17).

CDK2 has a pivotal role in facilitating tumorigenesis. As a key regulator of multiple oncogenic signaling pathways, its activity is important in the unregulated cell cycle and proliferation (18–22). CDK2 is implicated not merely in the cellular cycle process, but the inhibition of CDK2 within tumor cells is also instrumental in eliciting anti-neoplastic immune reactions (3, 23). Other CDKs also play an important role in anti-tumor immunity (24–27).

CDKs inhibitors combined with chemotherapy drugs can enhance anti-tumor efficacy (28–30). Although chemotherapeutic agents are known to induce ICD, the efficacy exhibits variability. Combining anthracyclines with CDK2 inhibition presents a therapeutic strategy, leveraging the immunostimulatory properties of both. The concomitant administration of chemotherapy with CDK2 inhibitor elicited a more potent therapeutic pathway compared to chemotherapy as a sole treatment modality (31). Our findings indicate that *Cdk2^-/-^* tumor cells combined anthracyclines trigger a more robust series of immunostimulatory stress pathways than WT cells, including apoptosis stress, endoplasmic reticulum stress, HMGB1 release, and type-1 interferon response, stimulate DCs and T cells infiltration. These responses collectively foster a protective anticancer immune reaction.

1Many studies indicated that ICD-based immunotherapy, that was chemotherapy drugs combined with anti-PD-1/PD-L1 antibodies, significantly enhances anti-tumor efficacy (32–35). Also, CDKs inhibitor can enhance PD-L1 protein expression and programmed cell death protein 1 immune checkpoint blockade efficacy (24, 29, 36). Advances in understanding ICD have revealed that inhibitor of CDKs, Dinaciclib, can modulate cancer cell immunogenicity and augment the overall efficacy of anti-PD-1 checkpoint blockade (3, 37). Nevertheless, Dinaciclib, which is a selective CDK inhibitor targeting CDK1, CDK2, CDK5, and CDK9, appears to be insufficient in conclusively demonstrating the role of CDK2 in tumor immunogenicity (3, 38) and a role for the specific inhibition of CDKs in inducing ICD has not been described. Here, we demonstrate that *Cdk2^-/-^* tumor cells not only sensitize MTX-induced ICD to drive the expression of ICD hallmarks, stimulate immune cell infiltration, and inhibit tumor growth but also enhance the effects of anti-PD-1 therapy to provoke a robust anticancer immune response, result in enhanced anti-tumor immunity, culminating in tumor regression, suggesting a synergistic therapeutic relationship.

## Materials and methods

### Cell culture

Cells were cultured in DMEM (Solarbio, Cat 11995), supplemented with 10% FBS (Cat 10099141) and 100 U/mL penicillin and streptomycin (Cat 15140122) from Thermo Fisher Scientific. Cells were incubated at 37°C in a humidified atmosphere containing 5% CO2.

### *Cdk2-/-* cancer cell line generation and transfection

*Cdk2^-/-^* MCA205 cells were generated by synthesizing sgRNA sequences targeting *Cdk2* and cloning them into the LentiCRISPR V2 vector (Feng Zhang’s gift, Addgene plasmid #52961). Lentivirus was produced in 293T cells through co-transfection with pMD2.G (Addgene plasmid #12259), psPAX2 (Addgene plasmid #12260), and either the *Cdk2* or control vector plasmid. Virus supernatant was collected 48 hours post-transfection, filtered through a 0.45 μm filter, and stored at -80°C or used immediately. After 48 hours of infection, cells were cultured in puromycin selection medium (4 μg/ml) for at least 7 days. Monoclonal cells in 96-well plates were obtained using the FACSAria™ III cell sorter (Becton Dickinson, San José, CA, USA). sgRNA sequences: F:5’-CACCGTTGTGGCGCTTAAGAAGATC-3’; R:5’- AAACGATCTTCTTAAGCGCCACAAC-3’.

### Overexpression vectors and transfection

For CD39 overexpression, the dCAS9-CD39_GFP plasmid was constructed with GENEWIZ’s assistance. The dCAS9-VP64_GFP plasmid (Addgene Cat #61422) was digested with BamHI and NheI, replacing the VP64 sequence with CD39 cDNA obtained via gene synthesis. 293T cells were transfected with the dCAS9-VP64_GFP plasmid, packaging plasmid psPAX2 (Addgene Cat #12260), and envelope pMD2.G (Addgene Cat #12259) using Lipofectamine 3000 following the manufacturer’s instructions. Lentivirus was harvested from the cell culture medium (DMEM+10% FBS) after 48 hours, collected by centrifugation, filtered through a 0.45 μm filter, and stored at -80°C or used immediately. *Cdk2^-/-^* MCA205 cells were infected with CD39 lentivirus for 48 hours, and infected positive cells were isolated by fluorescence-activated cell sorting. For CDK2 overexpression, a dCAS9-CDK2_GFP plasmid was constructed with GENEWIZ’s help (*Cdk2* cDNA obtained via gene synthesis). The CDK2 sgRNA base sequence in the dCAS9-CDK2_GFP plasmid was altered without changing the encoded amino acid to avoid CRISPR CAS9 enzyme cleavage (5’TGTGGCGCTTAAGAAGATCCGGCTCGACACT3’ to 5’TAGTCGCCCTAAAAAAAATAAGATTGGATACGG3’). For mutant CDK2 (T160A) overexpression, a dCAS9-CDK2^T160A^_GFP plasmid was constructed with GENEWIZ’s assistance, altering the CDK2 sgRNA base sequence without changing the encoded amino acid.

### Tumor models

Animal experimental protocols received approval from the Guizhou Medical University Animal Ethics Committee. Naïve female C57BL/6N and athymic nude BALB/c mice (Nu/Nu) were obtained from Beijing Vital River Company. *Tlr4^-/-^* C57BL/6 mice, aged 6–8 weeks, were sourced from the Model Animal Research Center of Nanjing University. Mice were randomly assigned to designated groups (5–10 mice per group) prior to inoculation. Tumor cells (2×10^6 cells in 100 μl PBS per mouse) were subcutaneously implanted in C57BL/6N mice or 1×10^6 cells μl PBS per mouse for Nu/Nu mice. Chemotherapy via intraperitoneal injection of MTX (1 mM, 200 μl) administered in three doses (days 5, 7, 10 or days 7, 9, 11, it depends on the tumor sizes at days 5). When necessary, mice received intraperitoneal injections of blocking antibodies (200 μg per injection) against IFNAR (InVivoMAb, Clone: MAR1-5A3, BioXcell) on days 0, 3, 6, 9, 12, 14, 18, or intravenous injections of blocking antibodies against PD-1 (InVivoMAb, Clone: RMP1-14, BioXcell) on days 7, 9, 11, 13, 15, 17. All mice were euthanized by CO2 asphyxiation. Tumor growth was monitored through periodic caliper measurements, calculated as the product of tumor length and width. Animals were euthanized when tumor volume reached 300 mm². Tumor progression was tracked 2–3 times per week and presented as error bars of mean ± SEM at each time point.

### Western blot

MCA205 WT cells were treated with 1 μM MTX or 20 ng/ml TNFα for 24h. Cells were treated or not as described and lysed in lysis buffer (Cat P0013, Beyotime) and protein concentration was determined using BCA Protein Assay Kit (Cat P0011, Beyotime). Equal amounts of total protein were separated by SDS-PAGE gels (10% or 12.5%) and transferred to 0.45 μm PVDF membranes. Membranes were blocked with 5% non-fat milk in TBST. Then the membranes were incubated with specific primary antibodies: PARP (catalog 9542S), FLIP (catalog 56343S), CASPASE-3 (catalog 9665S), Cleaved-CASPASE-3 (catalog 9664S), CASPASE-8 (catalog 4790S), Cleaved-CASPASE-8 (catalog 8592S), β- TUBULIN (catalog 2146T); Subsequently, incubated with second antibodies: Anti-mouse IgG, HRP-linked Antibody (catalog 7076) or Anti-rabbit IgG, HRP-linked Antibody (catalog 7074) (Cell Signaling Technology). The membranes were scanned with the ChemiDoc XRS+ system (Bio-Rad, USA).

### qRT-PCR

WT and *Cdk2^-/-^* MCA205 cells were treated with 1μM MTX for 24h. After total cellular RNA was extracted, RNA concentration was determined using the Nanodrop machine and software (Thermo Fisher Scientific). A total of 4 μg RNA was used to generate cDNA with PrimeScript™ II 1st Strand cDNA Synthesis Kit for qRT-PCR (Cat 6110A, TaKaRa). *Cxcl10, Gbp4, Ccl7, Rasd2, Parp14, Oasl2* and *Gapdh* relative copy number were determined by calculating the fold change difference in the gene of interest relative to *Gapdh.* qRT-PCR was performed on the Applied Biosystems 7500 machine. All the sequences used in the present study were listed in **Supplementary Table S1**.

### Dataset analysis

The method of correlation analysis is the Spearman rank correlation coefficient. Statistical analyses and data visualization were conducted utilizing R software, specifically version 3.6.3. The R package employed in this research is GSVA, version 1.34.0, as described by Hänzelmann et al. (2013). The algorithm for immune infiltration within the study is ssGSEA, which is integrated within the GSVA package. The focal disease under investigation is Sarcoma. The dataset utilized in this analysis is derived from The Cancer Genome Atlas (TCGA), accessible at https://portal.gdc.cancer.gov/, specifically the SARC project level 3 HTSeq-FPKM formatted RNA sequencing data. In terms of data transformation, the RNA sequencing data, initially in FPKM format, was converted to TPM (Transcripts Per Million) format and subsequently log2 transformed to normalize the data distribution. Data filtering in this study involved the exclusion of control and normal samples, for that not all projects encompass control/normal samples. To further augment the dataset, markers indicative of 24 distinct immune cell types were sourced from a publication in *Immunity* by Bindea, Gabriela, et al. (2013), which provides a comprehensive delineation of cell classifications and descriptions. Assessment of normality was undertaken using the Shapiro-Wilk test, while variance homogeneity was evaluated via Levene’s test. The P-values were determined employing an independent samples CDK2 expression data in patients treated with nivolumab were obtained from the GEO dataset GSE91061. A publication in *Cell* by Nadeem Riaz et al. (2017), which provides a comprehensive description of patient samples.

### ATP release quantification

WT and *Cdk2^-/-^* MCA205 cells were seeded at 3×10^4 cells per well in a 24-well plate. The following day, the medium in all wells was replaced with pre-warmed fresh medium (300 μl per well), with or without 2 μM MTX. After 24 hours, supernatants were collected, centrifuged at 500×g to remove cell debris, and used immediately to measure ATP release using the ENLITEN^®^ ATP Assay System (Promega, FF2000, Madison, WI, USA). Chemiluminescence signals were measured using a VICTOR™ X multilabel reader (PerkinElmer).

### Cell death, calreticulin exposure and HMGB1 release

WT and *Cdk2^-/-^* MCA205 cells were seeded at 3×10^4 cells per well in a 24-well plate. The following day, the medium in all wells was replaced with pre-warmed fresh medium (300 μl per well), with or without 1 μM MTX. Collected cells and supernatants every 8 hours. Early or late apoptosis was analyzed using DAPI and Annexin V staining (BD Pharmingen™, Le Pont-De-Claix, France) following the manufacturer’s instructions. Calreticulin exposure was evaluated by surface staining using a rabbit monoclonal antibody (dilution 1:500, clone EPR3924, Ref: AJ1124a, ABGENT), followed by Alexa 488-conjugated goat anti-rat IgG (H+L) secondary antibody (dilution 1:500, Life Technologies). For the HMGB1 release assay, cell lines were seeded and treated with 1 μM MTX under conditions identical to the ENLITEN^®^ ATP Assay. Supernatants were collected after 24 hours, centrifuged to remove cell debris, and used immediately for ELISA-based quantifications (IBL International GMBH, Ref ST51011). Plates were read using a VICTOR™ X multilabel reader (PerkinElmer).

### Statistical analyses

All results are presented as means ± SEM from n = 3 to 6 parallel assessments, with similar results observed in at least two independent experiments. Statistical differences were evaluated using unpaired, two-tailed Student’s t-test or Mann-Whitney U test. Statistical analyses and histograms were generated using GraphPad Prism 5 software (San Diego, CA, USA).

## Results

### *Cdk2-/-* tumor cells are sensitive to chemotherapy and dependent on the immune system

**Figure 1:**
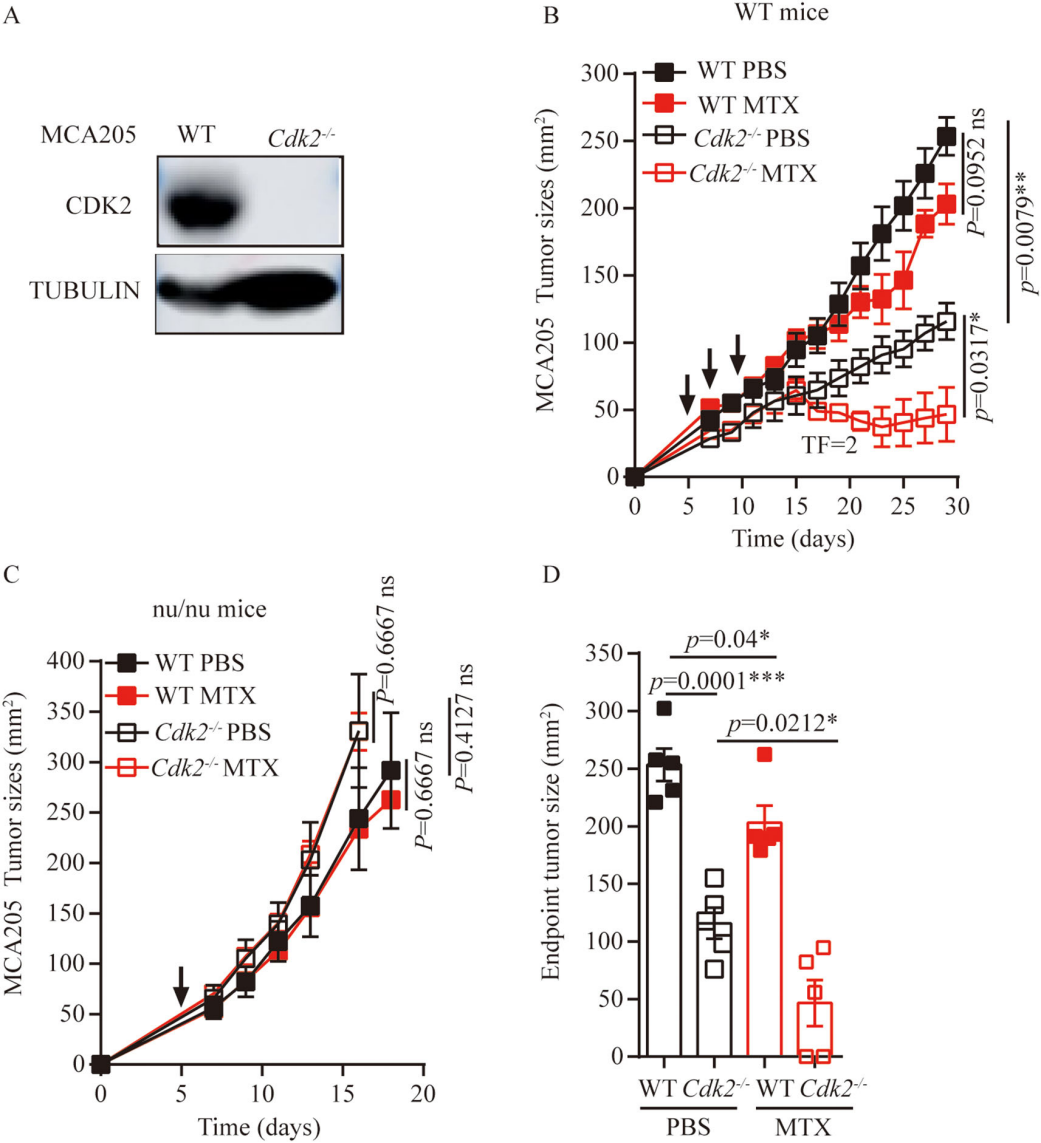
*Cdk2^-/-^* MCA205 cells enhance the therapeutic efficacy of MTX through an immune-dependent mechanism. **(A)**, Western blot showing expression of indicated proteins in WT, *Cdk2^-/-^* MCA205 cells. **(B)**, Immunocompetent mice bearing WT or *Cdk2^-/-^* MCA205 fibrosarcomas received intraperitoneal injections of PBS or MTX (1mM/200 μL/mouse). **(C)**, Athymic nude mice (nu/nu) with WT or *Cdk2^-/-^* MCA205 fibrosarcomas were treated as descried. **(D)**, Tumor sizes at 29 days were compared among MTX treated cancers with the indicated genotype. Tumor sizes, monitored 2–3 times weekly, were presented as mean ± SEM at each time point. Each group comprised 5–6 mice, with experiments repeated at least twice. Tumor growth curve differences were analyzed using the nonparametric Mann-Whitney U test, while endpoint tumor sizes differences were analyzed using the unpaired Student’s t-test; asterisks denote statistical significance (*p<0.05, **p<0.01, ***p<0.001, ns, nonsignificant). Arrows indicate dosing dates (days 5, 7, 10). TF, tumor free.

We used CRISPR-Cas9 technology to generate *Cdk2^-/-^* MCA205 fibrosarcomas cells that lacked *Cdk2* expression. We have demonstrated through the western blot method that CDK2 protein is not expressed in *Cdk2^-/-^* cells (**Figure 1A**). In response to the anthracycline analogue MTX, *Cdk2^-/-^* MCA205 cells exhibited a stronger chemotherapeutic response to MTX *in vivo* than WT cells (**Figure 1B**). However, the enhanced chemotherapeutic response of *Cdk2^-/-^* MCA205 cells was not observed in T cell-deficient nu/nu mice (**Figures 1C, D**). Despite the fact that *Cdk2^-/-^* MCA205 tumors grew slower than WT, *Cdk2^-/-^* tumors after MTX treatment were statistically different from tumors without chemotherapy. There was no difference between the WT tumor curves with or without chemotherapy (**Figure 1B**) using unpaired Mann-Whitney U test, while there was difference between the WT endpoint tumor sizes with or without chemotherapy using unpaired Student’s t-test (**Figure 1D**). These results suggest that depletion of *Cdk2* enhances the efficacy of anthracyclines in an immune dependent fashion.

### Enhanced local immune response of *Cdk2^-/-^* tumors responding to anthracyclines

**Figure 2:**
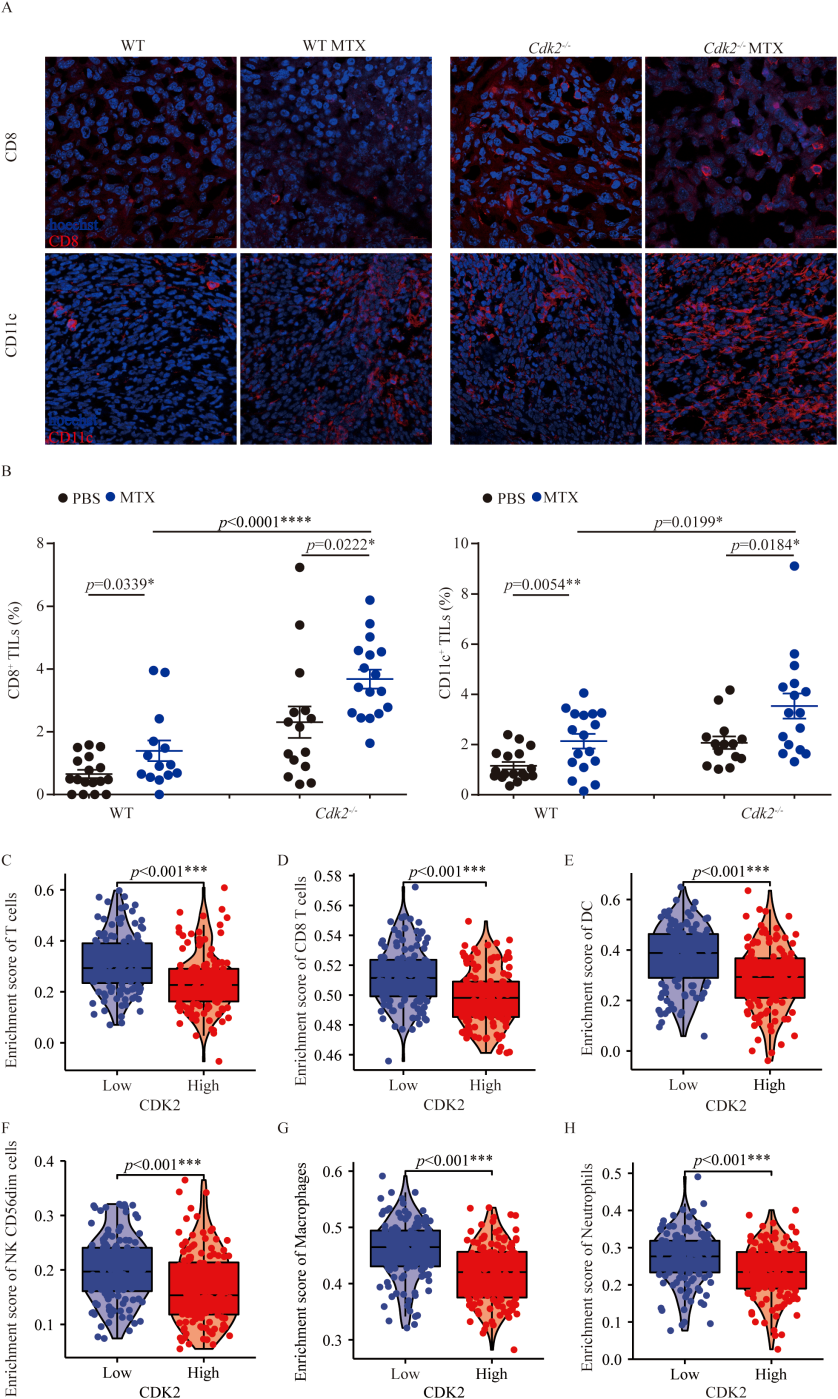
*Cdk2* deletion in fibrosarcoma promotes immune cell infiltration after MTX treatment. Mice with WT or *Cdk2^-/-^* tumors were injected (i.p.) with PBS or MTX. Tumor samples, collected 48 hours post-treatment, underwent immunofluorescence staining. Representative images from MTX-treated tumors were shown in **(A)**. Tumor-infiltrating CD8^+^ cells (left) and CD11c^+^ cells (right) were quantified as a percentage of all nucleated (Hoechst+) cells in the same field. Enrichment of T cells **(C)**, CD8^+^ T cells **(D)**, DC **(E)**, NK cells **(F)**, Macrophages **(G)**, and Neutrophils **(H)** was assessed in low versus high CDK2 expression in sarcomas patients. The GSVA package in R [version 1.34.0] calculated immune infiltration in sarcomas (control/normal removed) from TCGA, with RNAseq data in HTSeq-FPKM format converted to TPM format and log2 transformed. Normality (Shapiro-Wilk test) and variance homogeneity (Levene’s test) were evaluated, and P-values were determined by independent samples T-test. Each group contained 5 tumor samples, each point representing a different field. Non-consecutive sections from the same tissue were randomly selected, images captured from both tumor centers and peripheries. *p<0.05, **p<0.01, ***p<0.001, ****p<0.0001 (unpaired Student t test).

As mentioned above, *Cdk2^-/-^* tumors growing smaller on immunocompetent mice than WT tumors in response to MTX treatment. Therefore, we analyzed the difference in immune cell infiltration in the tumor microenvironment between WT tumors and *Cdk2^-/-^* tumors after MTX treatment. And we found that *Cdk2^-/-^* tumors exhibited a major alteration on their immune cell infiltration. Specifically, within 48 hours post-chemotherapy, *Cdk2^-/-^* tumors manifested a denser infiltration by CD8^+^ cells (**Figures 2A, B**) than did control tumors. Similarly, the frequency of CD11c^+^ cells (**Figures 2A, B**) increased by immunofluorescence, followed by microscopic quantitation. Moreover, through analysis of TCGA, we found that sarcoma patients with low expression of CDK2 had higher enrichment scores of T cells, DC, NK cells, macrophages and neutrophils (**Figures 2C–H**). Altogether, these results suggest that the combination of MTX plus *Cdk2* depletion can stimulate a particularly immune-mediated chemotherapeutic response.

### *Cdk2^-/-^* cancer cells are more susceptible to apoptosis responding to MTX

MTX has its ability to induce cytotoxicity and immunogenic cell death (8, 39).

**Figure 3:**
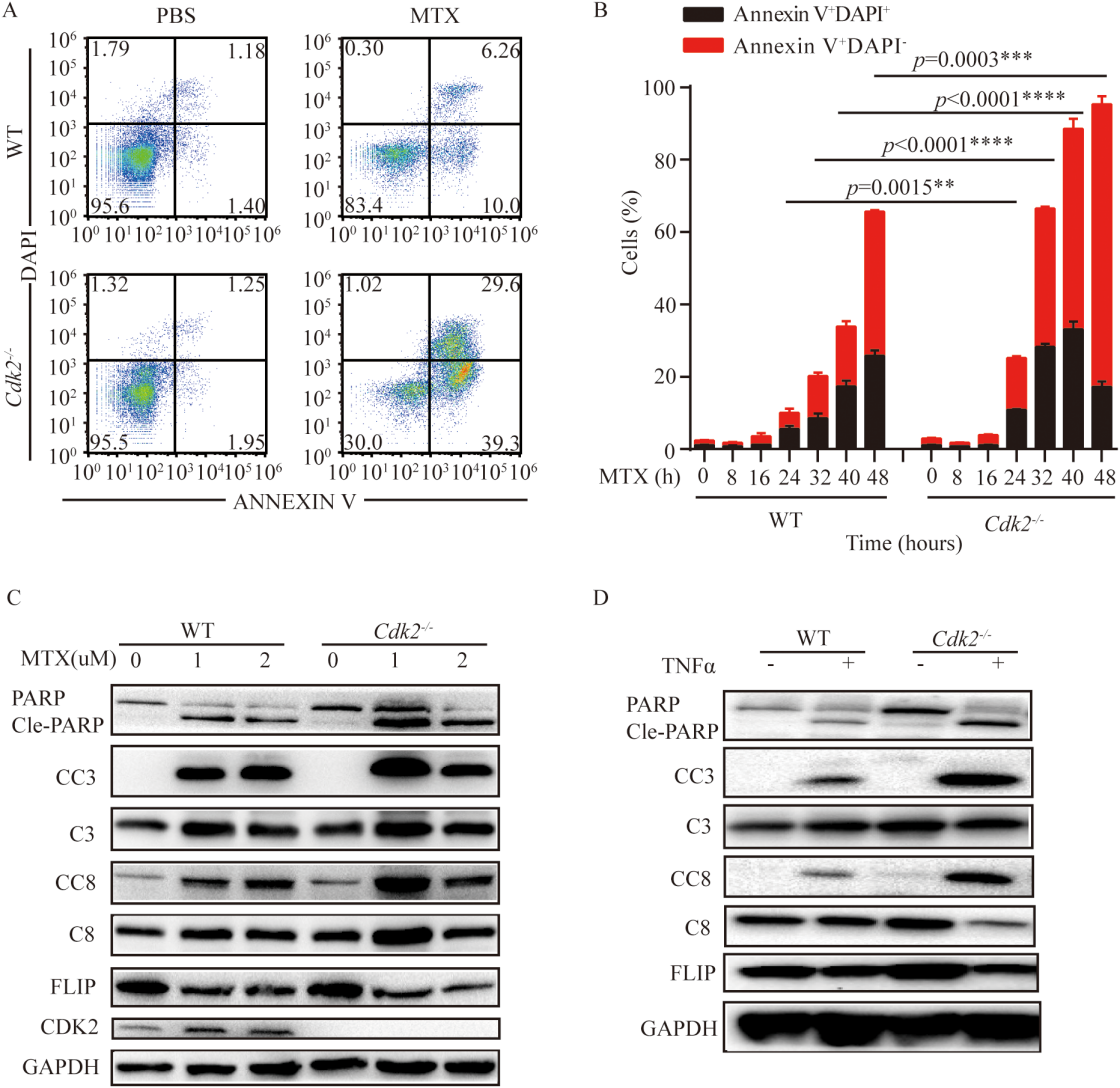
*Cdk2^-/-^* cells were more prone to apoptosis than WT cells after MTX treatment. **(A, B)** WT and *Cdk2^-/-^* MCA205 cells were treated with MTX *in vitro* using 6 well plates. Cells were collected at specified times to assess apoptosis via Annexin V-FITC and DAPI staining. Typical dot plots are presented at 24 h **(A)**, the mean ± SEM (n=3 independent experiments) of percentages of cells in early and late apoptosis (Annexin V^+^ DAPI^+^) were plotted **(B)**. **(C, D)** WT and *Cdk2^-/-^* cells were treated with or without MTX **(C)** or TNFα **(D)** for 24 hours, then western blot analysis of apoptosis-related proteins in WT and *Cdk2^-/-^* cells was conducted. Experiments were repeated at least twice. **p<0.01, ***p<0.001, ****p<0.0001 (unpaired Student’s t test). PARP, Poly (ADP-ribose) polymerase; Cle-PARP, Cleaved PARP; CC3, Cleaved Caspase-3; C3, Caspase-3; CC8, Cleaved Caspase-8; C8, Caspase-8; FLIP, FLICE/caspase8-inhibitoryproteins; CDK2, Cyclin-dependent kinase 2; GAPDH, Cyclin-dependent kinase 2.

As mentioned above, *Cdk2^-/-^* tumors are more sensitive to MTX and can promote the infiltration of immune cells into the tumor microenvironment. We found that *Cdk2^-/-^* MCA205 cells were more prone to apoptosis than WT after treatment with MTX. We noticed that the proportion of early apoptosis (AnnexinV^+^ DAPI^-^) and late apoptosis (AnnexinV^+^ DAPI^+^) in *Cdk2^-/-^* cells increased after MTX treatment (**Figures 3A, B**), and the representative flow scatter plot is shown in **Figure 3A**. Similarly, we found that cleaved-caspase and cleaved-PARP proteins, which promote apoptosis, exhibited high expression and FLIP, which inhibits apoptosis, exhibited low expression in *Cdk2^-/-^* MCA205 cells after treatment with MTX or TNFα by western blot (**Figures 3C, D**).

### Essential role for the type I IFN pathway in the improved chemotherapeutic response of *Cdk2^-/-^* tumors

**Figure 4:**
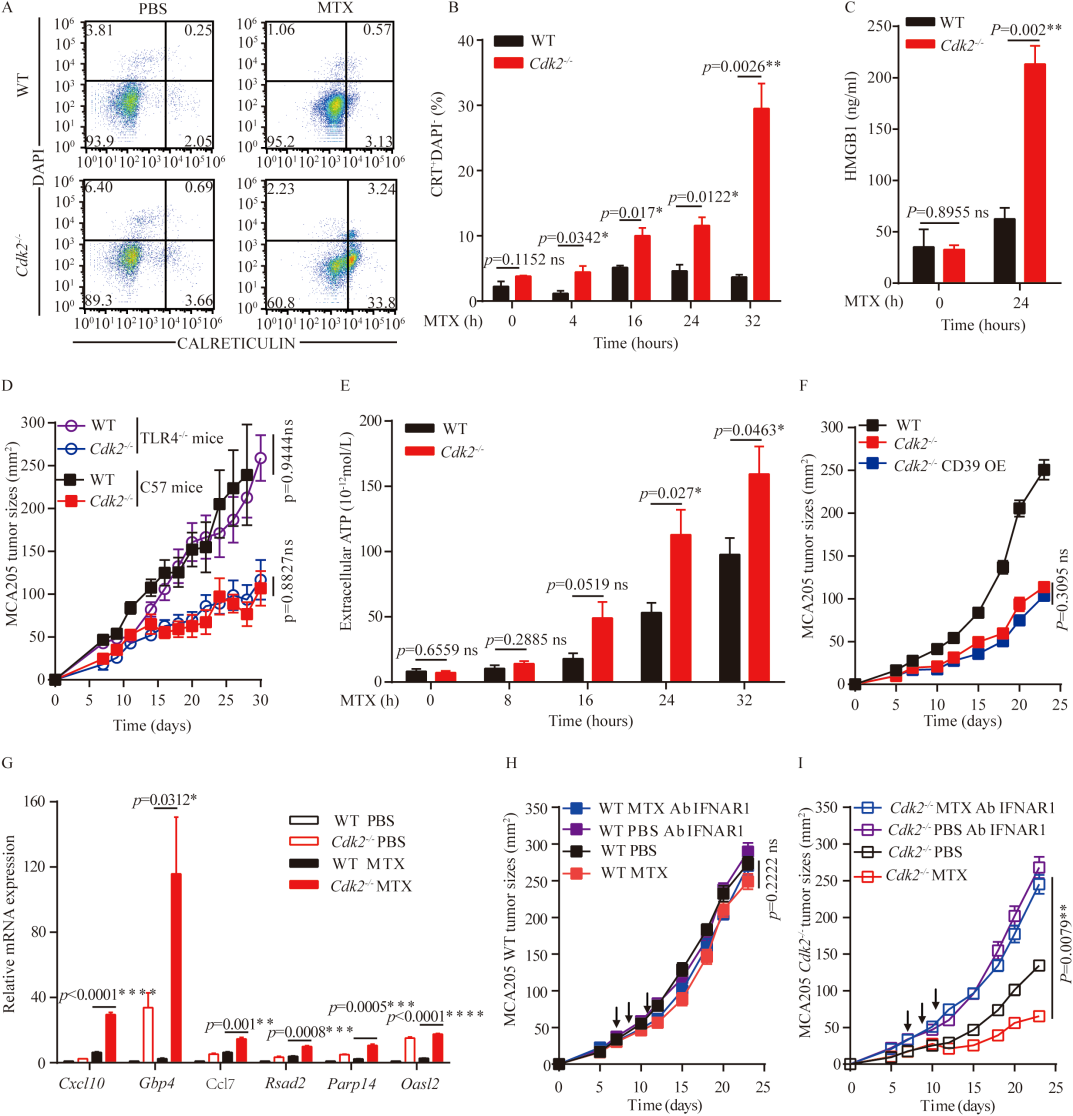
Impact of *Cdk2* deficiency on the hallmarks of immunogenic cell death. **(A, B)** WT and *Cdk2^-/-^* MCA205 cells were treated with MTX *in vitro* in 6-well plates, and at the indicated time points, cells were collected to assess surface calreticulin exposure using rat monoclonal anti-Calreticulin antibody. Typical dot plots **(A)** and quantitative data **(B)** are presented. **(C)** HMGB1 protein levels in supernatants from WT or *Cdk2^-/-^* cells pre-treated with MTX for 24 hours were measured by ELISA. **(D)** Growth curves of immunocompetent mice bearing WT, *Cdk2^-/-^*, or CD39 overexpressing *Cdk2^-/-^* MCA205 tumors are shown. **(E)** Supernatants from WT or *Cdk2^-/-^* MCA205 cells treated with MTX at different times were collected to quantify ATP release using the luciferase bioluminescence assay. (F) Tumor growth in mice bearing MCA205 cells with WT, *Cdk2^-/-^*, or *Cdk2^-/-^* CD39-overexpression genotypes. **(G)** Type I IFN-related gene expression, with or without MTX treatment for 16 hours, was measured in WT and *Cdk2^-/-^* tumor cells. Relative expression levels compared to untreated WT MCA205 cells were calculated using the 2^-ddCT method and expressed as fold-change. **(H, I)** WT **(H)** or *Cdk2^-/-^* **(I)** MCA205 tumor cells were inoculated into immunocompetent mice, mice received injections of PBS, MTX, or neutralizing IFNAR1 antibodies (i.p.). Arrows indicate the timing of MTX injections. All groups contained 5 mice, with experiments repeated twice. For gene expression data, unpaired Student’s t-test was applied. Tumor growth differences were assessed using the unpaired Mann-Whitney U test. *p<0.05, **p<0.01, ***p<0.001, ****p<0.0001, ns, not significant. Arrows indicate dosing dates (MTX treatment at days 7, 9, 11).

MTX can stimulate a series of stress and death pathways, including apoptosis, exposure of calreticulin upon endoplasmic reticulum stress, nuclear exodus of HMGB1 resulting from secondary necrosis, autophagy-linked ATP release, and the production of chemokines (such as CXCL10) subsequent to type-1 interferon response to induce immunogenic cell death. Since MTX is an inducer of immunogenic cell death, we analyzed the changes in immunogenic cell death markers after MTX treatment at the cellular level. Accordingly, calreticulin exposure could be induced by MTX in *Cdk2^-/-^* MCA205 cells, moreover *Cdk2^-/-^* cells exhibited an increased calreticulin exposure after 4 hours (**Figures 4A, B**). Similarly, *Cdk2^-/-^* MCA205 cells cultured with MTX released more HMGB1 than WT cells (**Figure 4C**). Since HMGB1 can be recognized by TLR4 on immune cells and activate anti-tumor immunity, we used *Tlr4^-/-^* mice to block extracellular HMGB1 triggered antitumor immunity. We subcutaneously implanted WT and *Cdk2^-/-^* cancer cells into C57 mice and *Tlr4^-/-^* mice respectively. We found that tumors grew slower with *Cdk2^-/-^* cells than those with WT cells in both C57 and *Tlr4^-/-^* mice, which suggested that HMGB1-TLR4 signaling is dispensable for *Cdk2^-/-^* cells increased immunogenicity (**Figure 4D**).

ATP is a key damage-associated molecular pattern in ICD in addition to HMGB1 and CRT. We found that *Cdk2^-/-^* MCA205 cells released more ATP than WT cells in response to MTX (after 24 hours), as indicated by the measurement of extracellular ATP (**Figure 4E**). The increase of ATP release in *Cdk2^-/-^* cells and enhanced dendritic cells infiltration in *Cdk2^-/-^* tumors suggested that ATP might play a critical role in antitumor immunity in *Cdk2^-/-^* tumors. To abolish extracellular ATP in tumor microenvironment, we overexpressed the ATP hydrolase CD39 protein in *Cdk2^-/-^* cells, and inoculated the obtained stable cell lines into C57 mice subcutaneously. The tumor growths of *Cdk2^-/-^* and *Cdk2^-/–^* CD39 overexpression cells were similar and slower than those of WT MCS205 cells, which suggest that their differences in ATP release might not be responsible for their differential tumor growth rates (**Figure 4F**). More importantly, we demonstrated that *Cdk2* deficiency increased the expression of ISGs than WT in response to MTX treatment (**Figure 4G**), as determined by qRT-PCR analyses. These upregulated ISGs (Interferon-Stimulated Genes) included the antiviral genes *Parp14*, a number of other type-1 interferon-induced genes (such as *Gbp4*, *Oasl2* and *Rsad2*), as well as to the chemokine-encoding gene *Cxcl9* and *Ccl7*, which code for CXCR3 ligands and CCR ligands respectively (**Figure 4G**). To further determine if the interferon signaling pathway is involved in MTX-mediated anti-tumor effects in *Cdk2^-/-^* tumors, we treated C57 mice carrying *Cdk2^-/-^* MCA205 tumors with antibodies that neutralize IFNAR1. We found that antibodies reduced the efficacy of chemotherapy against *Cdk2^-/-^* tumors and also eliminated the difference in the therapeutic response between WT and *Cdk2^-/-^* tumors (**Figures 4H, I**). Together, our above studies indicate that the increase of MTX-induced immunogenicity in tumors carrying *Cdk2^-/-^* cancer cells were dependent upon the elevated type-1 interferon response.

### CDK2 kinase activity is involved in the enhanced chemotherapeutic response of *Cdk2^-/-^* tumors

**Figure 5:**
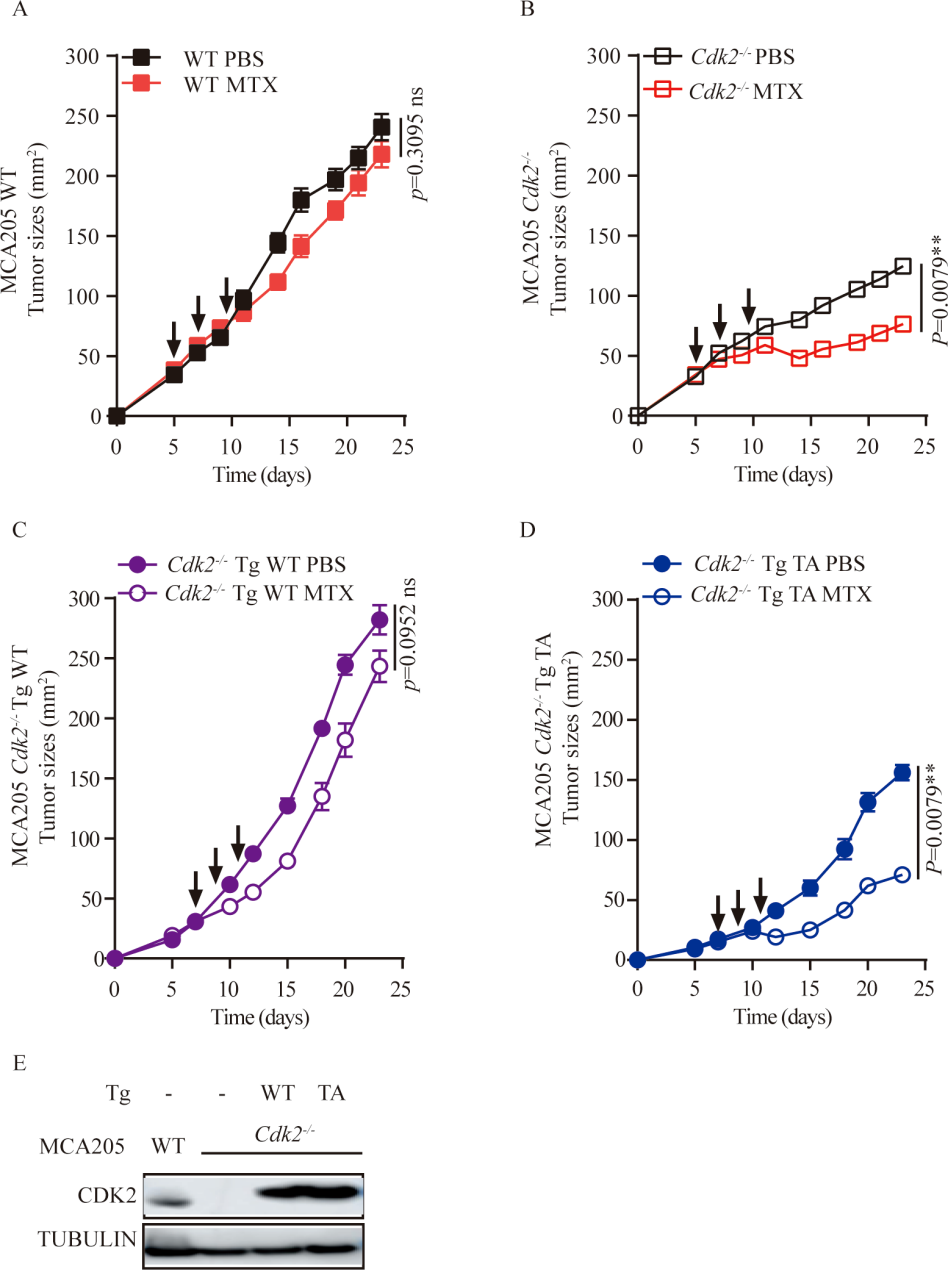
Re-introduction of WT, but not mutant *Cdk2* abolishes the therapeutic advantage of *Cdk2^-/-^* tumors. **(A-D)** WT or mutated (T160A) forms of *Cdk2* were stably transfected into *Cdk2^-/-^* MCA205 cells. The responsiveness of *Cdk2^-/-^* Tg WT **(C)** or *Cdk2^-/-^* Tg T160A **(D)** to chemotherapy was compared to that of WT **(A)** and *Cdk2^-/-^* **(B)** tumors, in immunocompetent mice. Arrows indicate the time when MTX was administrated. **(E)** Validation of CDK2 expression upon re-introduction of WT or mutant *Cdk2* into *Cdk2^-/-^* cells. All groups included at least 5 mice and one representative experiment out of two is shown. **p<0.01, ns, not significant (unpaired Student t test).

If CDK2 inhibited the local immune response by virtue of its function as a kinase protein, reconstitution of *Cdk2^-/-^* cancer cells with WT but not the kinase inactive T160A mutant *Cdk2* should reverse the phenotype. Accordingly, we found that the enhanced MTX anti-tumor responses of *Cdk2^-/-^* cancer cells over WT cells were abolish with WT *Cdk2* but not *Cdk2* ^T160A^ reconstitution (**Figures 5A–D**). Validation of CDK2 expression was confirmed through Western blot analysis following re-introduction of WT or mutant Cdk2 into Cdk2^-/-^ cells (**Figure 5E**). These results indicate that CDK2 kinase activity have essential role in the anti-tumor effects of MTX.

### *Cdk2* deletion is sensitive to anti-PD-1 therapy

**Figure 6:**
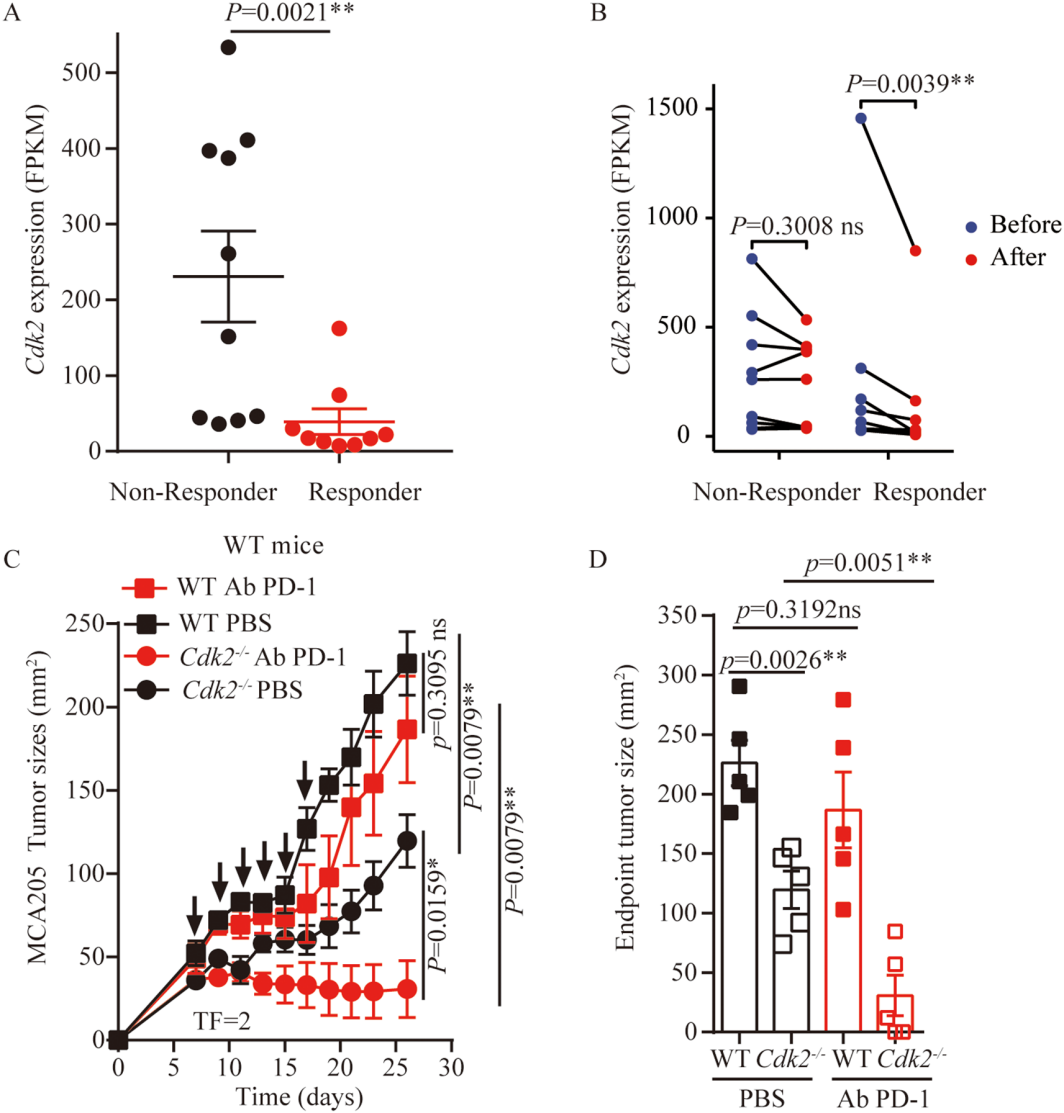
*Cdk2^-/-^* tumors combined with anti-PD-1 treatment exhibited better outcome than WT tumors. **(A)** Expression of CDK2 in patients with melanoma who benefited from nivolumab treatment and those who did not benefit, data from GSE91061. **(B)** CDK2 expression is reduced in people who respond to anti-PD-1 therapy, data from GSE91061. **(C)** WT tumors’ responsiveness to anti-PD-1 antibodies (i.v) was compared with *Cdk2^-/-^* tumors in immunocompetent mice, arrows marking anti-PD-1 antibody administration. **(D)** Tumor sizes at 26 days were compared among anti-PD-1 antibodies treated cancers with the indicated genotype. Each group included at least 5 mice. For *Cdk2* gene expression, the Mann-Whitney test and Wilcoxon rank test were used. Tumor growth comparisons employed the unpaired Mann-Whitney U test. Endpoint tumor sizes differences were analyzed using the unpaired Student’s t-test. Response categories included CR/PR/SD, with non-response as PD. *p<0.05, **p<0.01, ns, not significant.

Anti-PD-1 antibodies are commonly used clinically as immune checkpoint inhibitors. Many studies indicated that ICD-based immunotherapy, that was chemotherapy drugs combined with anti-PD-1/PD-L1 antibodies, significantly enhances anti-tumor efficacy (32–35). Also, CDKs inhibitor can enhance PD-L1 protein expression and programmed cell death protein 1 immune checkpoint blockade efficacy (24, 29, 36). First, we compared the expression of CDK2 in patients with advanced melanoma who responded to anti-PD-1 therapy and those who did not responded using publicly available dataset (GSE91061). We found that CDK2 expression was reduced in responding patients compared with non-responding patients (**Figure 6A**). At the same time, we analyzed that there was no statistical difference in the expression of CDK2 before and after anti-PD-1 treatment in the non-responsive patient group. However, there was a statistical difference in the expression of CDK2 before and after anti-PD-1 treatment in the responding patient group (**Figure 6B**). Therefore, we explored the effect of inhibiting CDK2 expression in combination with anti-PD-1 antibodies *in vivo* experiments in mouse models. We found that after tail vein injection of anti-PD-1 antibodies into mice bearing WT or *Cdk2^-/-^* cancer cells, the *Cdk2^-/-^* tumors plus anti-PD-1 Ab grow slower than *Cdk2^-/-^* PBS group (**Figures 6C, D**), but there was no statistical difference in WT tumors with or without anti-PD-1 Ab treatment (**Figures 6C, D**). These data demonstrate that suppressing CDK2 enhances the antitumor activity of anti-PD-1 antibody.

## Conclusion

Previous studies provided compelling evidence that the CDK inhibitors can synergize with anthracyclines to mediate antitumor effects in preclinical models (28–30), thereby highlighting a potential mechanism for combinatorial therapy. According to the result that the enhanced chemotherapeutic response of *Cdk2^-/-^* MCA205 cells was not observed in T cell-deficient nu/nu mice, which indicates that, in the context of MTX, inhibition of CDK2 enhances anti-tumor immune responses, which may dependent on host T lymphocytes. Additionally, there is increased infiltration of immune cells in the tumor microenvironment. *In vitro* experiments reveal that CDK2 inhibition promotes immunogenic cell death. Type I interferon response plays a crucial role in the effect of CDK2 inhibition combined with MTX treatment, and the anti-tumor effect of MTX plus *Cdk2* deletion is related to its kinase activity. Furthermore, CDK2 inhibition improves the efficacy of anti-PD-1 therapy.

Although literature reported that CDK inhibitors can promote immunogenic cell death, whether *Cdk2* deficiency amplified the induction of ICD by anthracyclines remain to be elucidated. However, our findings suggest that *Cdk2^-/-^* tumor cells can enhance ICD triggered by anthracyclines. Our observations indicate that anthracycline-based chemotherapy is more efficacious against tumors when combined with *Cdk2* deletion, suggesting a synergistic interaction between CDK2 inhibition and anthracyclines. This synergistic effect was observed in immunocompetent mice but not in nu/nu mice. These results emphasize the role of the immune system in mediating the therapeutic interaction between CDK2 inhibition and anthracycline chemotherapy. We also observed that there was more immune cell infiltration in tumors treated with MTX than without MTX, especially in *Cdk2^-/-^* tumors. The enhanced infiltration of dendritic cells and cytotoxic T lymphocytes in *Cdk2* ^-/-^ tumors post-chemotherapy indicates a robust immune response, which may be attributed to the decreased growth observed in these tumors. This decreased tumor growth may reflect the heightened activity of immune effectors in the tumor microenvironment, potentially priming the tumors for a more effective immune attack. Our data suggests that *Cdk2* deficiency does not directly augment the cytotoxic action of anthracyclines, but enhances the secretion and expression of molecules related with immunogenic cell death, including ATP, HMGB1, CRT, and the production of immunostimulatory CXCR3 ligands like *Cxcl10*, implying that the observed synergy is immune-mediated rather than a consequence of increased direct cytotoxicity. This finding is consistent with literatures suggesting that the immune system plays a critical role in the efficacy of chemotherapy (40–43).

Although an increased expression of ICD-related molecules is observed *in vitro*, we observe that the tumor growth of *Cdk2^-/-^* cells with increased HMGB1 release in *Tlr4^-/-^* mice remained similar to that in C57 mice. The tumor growth of *Cdk2^-/-^* tumor cells overexpressing CD39 is comparable to that of *Cdk2^-/-^* tumor cells. However, the application of interferon receptor-blocking antibodies revers this effect, the loss of the favorable interaction between CDK2 inhibition and anthracycline-based chemotherapy upon neutralization of the common type-1 interferon receptor, establishes a cause-effect relationship between the exacerbated type-1 interferon-related response and the efficacy of the combined treatment. These results suggest that the type-I interferon response play a crucial anti-tumor role when CDK2 inhibition is combined with chemotherapy. By subcutaneously implanting restoration of *Cdk2^-/-^* cancer cells with WT *Cdk2* or *Cdk2* ^T160A^ into C57 mice, we observed that the combination of *Cdk2* ^T160A^ cells with MTX remained more effective than the replenished *Cdk2^-/-^* cancer cells. These results suggest that CDK2 kinase activity is involved in the improved chemotherapeutic response of *Cdk2^-/-^* tumors. Additionally, we observed that mice implanted with *Cdk2^-/-^* cells exhibited a better response to anti-PD-1 combination therapy than those implanted with WT cells. Analysis of the dataset also revealed that patients with a good response to anti-PD-1 therapy showed reduced CDK2 expression. These phenomena may be attributed to heightened sensitivity to immunogenic cell death, increased ISG expression and PD-L1 expression in tumor cells after CDK2 inhibition.

In conclusion, our study reveals that CDK2 inhibition enhances the antitumor effects and induces ICD with anthracycline-based chemotherapy. The findings suggest that CDK2 inhibition, in combination with anthracyclines or anti-PD-1 therapy, may offer a therapeutic strategy in cancer treatment. In recent years, with the advancement of molecular technology, PROTAC-targeted protein degradation technology has gained widespread attention due to its high specificity (44, 45). There have also been numerous reports on PROTAC degraders targeting CDK2 (46–48). Further research is warranted to explore the detailed molecular mechanisms and to evaluate the clinical applicability of this combinatorial approach, particularly in tumors that are ‘addicted’ to CDK2, where the cell-autonomous and immunological effects of CDK2 inhibition may complement each other advantageously.

## Data Availability

The raw data supporting the conclusions of this article will be made available by the authors, without undue reservation.
